# Supplementary material for: Candida albicans Promotes the Antimicrobial Tolerance of Escherichia coli in a Cross-Kingdom Dual-Species Biofilm
Source: Microorganisms. 2022 Nov 3;10(11):2179. doi: 10.3390/microorganisms10112179 (PMC9696809; doi:10.3390/microorganisms10112179)
Supplement: Supplementary file 1 [file microorganisms-10-02179-s001.zip › microorganisms-1965196-supplementary.pdf]

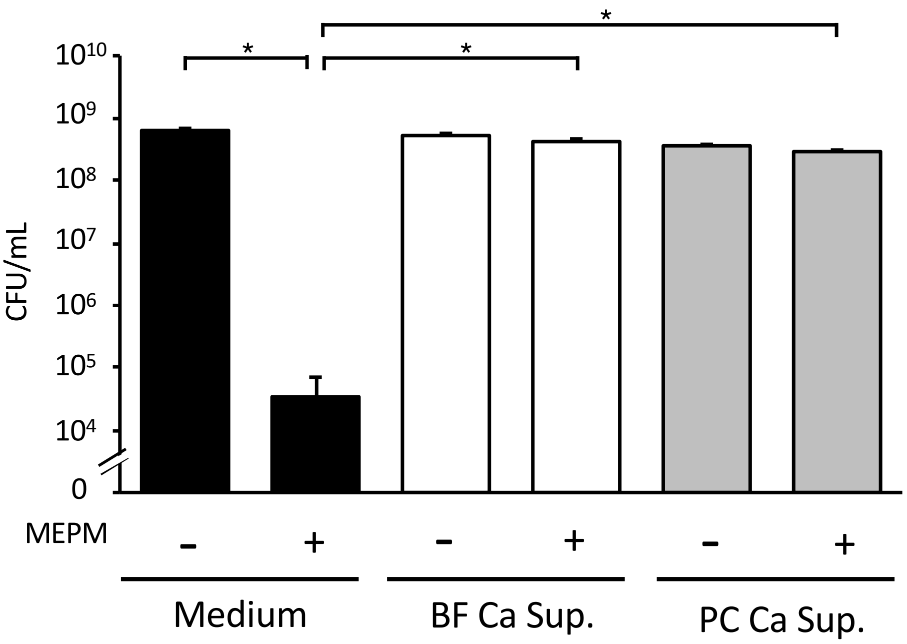

Figure S1
Effect of supernatants of *Candida* biofilms and planktonic cultures in the presence of MEPM.
Measurements were performed three times and the data are the means ± standard deviations. n = 3/group. Statistically significant differences between groups were evaluated using Tukey's test with one-way ANOVA; $^{*}P < 0.05$.
